# Association between health literacy and antibiotic-related knowledge, attitudes, and behaviors

**DOI:** 10.1186/s12889-026-26416-2

**Published:** 2026-01-26

**Authors:** Muhammet Salih Tarhan, Omer Guc

**Affiliations:** 1 Department of Infectious Diseases and Clinical Microbiology, Mardin Training and Research Hospital, Mardin, Türkiye; 2 Department of Family Medicine, Kocakoy State Hospital, Diyarbakır, Türkiye

**Keywords:** Health literacy, Antibiotic use, Antimicrobial resistance, Public health, Health behavior

## Abstract

**Background:**

Antimicrobial resistance (AMR) poses a growing threat to global health, driven by antibiotic misuse. Health literacy (HL) may play an independent role in shaping how people understand and use antibiotics. This study aimed to evaluate the relationship between HL and antibiotic-related knowledge, attitudes, and behaviors (KAB) to inform public health strategies that promote rational antibiotic use.

**Methods:**

A cross-sectional survey was conducted among 350 Turkish adults aged 18–65, using stratified quota sampling by age and gender. Data were collected via online forms and face-to-face interviews. Participants completed sociodemographic questions, the 6-item European Health Literacy Survey Questionnaire (HLS-EU-Q6), and a 16-item antibiotic-related KAB scale.

**Results:**

Of participants, 50% were female, mean age was 39.8 ± 12.2 years, and 62% had university education or higher. HL distribution was: inadequate 6.9%, problematic 78.6%, adequate 14.6%. Correlation analyses showed that HL was weakly associated with attitudes (*r* = 0.109, *p* = 0.041), behaviors (*r* = 0.150, *p* = 0.005), and total KAB (*r* = 0.130, *p* = 0.015), but not with knowledge. Knowledge was moderately correlated with attitudes (*r* = 0.307, *p* < 0.001) and weakly to moderately correlated with behaviors (*r* = 0.226, *p* < 0.001). Regression models indicated that attitudes were predicted by knowledge (β = 0.26, *p* < 0.001) and education (β = 0.16, *p* = 0.004), while behaviors were predicted by knowledge (β = 0.17, *p* = 0.002), HL (β = 0.14, *p* = 0.008), and age (β = 0.13, *p* = 0.023). Mediation/moderation revealed that HL and knowledge operated through independent pathways.

**Conclusions:**

Antibiotic knowledge was the strongest predictor of attitudes, while HL contributed independently to behaviors. These findings highlight the need for interventions that enhance both antibiotic knowledge and HL to promote rational use of antibiotics and prevent AMR.

**Supplementary Information:**

The online version contains supplementary material available at 10.1186/s12889-026-26416-2.

## Background

Antimicrobial resistance (AMR) is defined by the World Health Organization (WHO) as the ability of microorganisms such as bacteria, viruses, fungi, and parasites to resist the effects of antimicrobial agents, rendering standard treatments ineffective and increasing the risk of disease spread, severe illness, and death. According to the WHO, AMR puts at risk decades of progress in controlling infectious diseases and is projected to cause substantial morbidity, mortality, and economic burden globally [1]. The recent rise in AMR is largely attributed to the widespread misuse and overuse of antimicrobials [2]. Understanding the public’s knowledge, attitudes, and behaviors (KAB) regarding antibiotic use is therefore essential for developing effective AMR prevention and control strategies.

Health literacy (HL), which is defined as an individual’s ability to access, understand, evaluate and apply health information, plays an important role in shaping health-related decisions and behaviors [3]. Low HL has been associated with poor adherence to treatment, suboptimal health outcomes and increased misuse of medication [4]. While there is an increasing body of evidence to suggest that HL may influence medication-related behaviors, very few studies have specifically examined its impact on antibiotic use [5, 6].

Recent global evidence underscores a complex relationship between HL and antibiotic-related outcomes. In Europe, higher levels of HL are associated with greater citizen engagement in appropriate antibiotic use [7]. Similarly, evidence from the Middle East indicates that individuals with higher HL tend to demonstrate better understanding of AMR [8]. In Southeast Asia, population-based studies have reported that lower HL is linked to inappropriate antibiotic use and persistent misconceptions regarding antibiotic efficacy [9]. Collectively, these findings from diverse socio-cultural settings indicate that the relationship between HL and antibiotic-related KAB varies across populations.

From a conceptual perspective, HL may influence antibiotic-related outcomes through mechanisms that extend beyond factual knowledge acquisition. While knowledge reflects the understanding of specific information, attitudes and behaviors are additionally shaped by individuals’ ability to interpret health information, evaluate risks, and apply guidance in real-life decision-making contexts. Accordingly, HL may exert a differential influence on these constructs through partially distinct cognitive pathways—where information processing leads to knowledge, while evaluative skills are required for attitudinal and behavioral change. Within this conceptual framework, mediation and moderation analyses were used as exploratory tools to examine whether HL mediated or moderated the association between antibiotic knowledge and antibiotic-related attitudes and behaviors. These analyses were intended to characterise patterns of association rather than to imply causal pathways and were interpreted within the constraints of a cross-sectional design. Previous studies on antibiotic use have frequently focused on descriptive assessments of knowledge and awareness levels in the general population [10, 11]. However, the associations between HL and KAB related to antibiotics remain underexplored. Based on existing literature and the conceptual framework outlined above, the present study was guided by the following research expectations: (i) higher levels of HL would be associated with more appropriate antibiotic-related attitudes and behaviors; (ii) antibiotic knowledge would be positively associated with attitudes and behaviors; and (iii) HL and antibiotic knowledge would contribute independently to explaining variation in antibiotic-related behaviors. In line with this framework, we examined the associations between HL and antibiotic-related KAB among Turkish adults.

## Methods

### Study design and setting

We conducted a cross-sectional survey among adults aged 18–65. Participants were asked to provide sociodemographic data (sex, age, education, employment and chronic disease status) and to complete the 16-item KAB form and the 6-item European Health Literacy Survey Questionnaire (HLS-EU-Q6). Participants were recruited through open online invitations (Google Forms) and in-person administration at infectious diseases outpatient clinics at Mardin Training and Research Hospital. Participants from different regions of the country were included through an online survey. The Mardin region was also represented by a hospital-based sample. To maximise outreach and ensure a diverse sample, a hybrid recruitment strategy was adopted. The online survey was used to access a broad, geographically dispersed audience, while in-person recruitment at the infectious diseases clinic aimed to capture individuals who might be underrepresented in digital environments. To ensure the pooled data remained representative of the general adult population, and to mitigate potential selection bias from the different recruitment methods, strict stratified quota sampling based on national age and gender distributions was applied. Quota control was applied to the study, meaning that once a stratum reached its target, no more participants from that group were accepted. Data collection took place in September 2025.

### Sampling frame and stratification

To enhance the external validity of our findings and allow subgroup comparisons, we employed stratified quota sampling by age group and gender. Age strata were defined proportionally to the national population distribution (18–34: 37.0%, 35–49: 34.2%, 50–65: 27.8%), while gender was balanced at approximately 50:50 within each stratum.

### Pilot testing and reliability

Before the main survey, a pilot study was conducted with 34 participants to evaluate item clarity and to estimate reliability. Internal consistency was assessed using Cronbach’s alpha for each subscale KAB and HL, confirming their suitability for the main survey.

### Sample size and power

A pilot study with 34 participants revealed small correlations between HL and KAB outcomes (*r* ≈ 0.15–0.20). Based on these results, an a priori power analysis (G*Power 3.1) indicated that 240 participants were required to detect a small correlation (*r* ≈ 0.20) with 80% power (α = 0.05). The final sample consisted of 350 participants, providing sufficient power for the primary correlation and multivariable regression analyses. Mediation and moderation analyses using PROCESS were conducted on an exploratory basis and did not inform the sample size determination. Based on the calculated sample size and population weights, the planned quotas resulted in approximately 130 participants aged 18–34, approximately 120 participants aged 35–49, and approximately 100 participants aged 50–65.

### Data collection tools

#### Health literacy scale

HL was assessed using the short-form HLS-EU-Q6, derived from the original 47-item version (HLS-EU-Q47). The tool consists of six items rated on a 4-point Likert scale reflecting perceived difficulty (1 = very difficult; 2 = quite difficult; 3 = quite easy; 4 = very easy). The total score was divided by 6 to calculate the arithmetic mean. Based on this result, individuals’ HL levels were classified as follows: inadequate (score ≤ 2), problematic (score > 2 and ≤ 3) or adequate (score > 3). The Turkish version of the HLS-EU-Q6 has consistently demonstrated acceptable levels of psychometric reliability and validity (Cronbach’s alpha = 0.82) [12].

#### KAB questionnaire related to antibiotic use

The 16-item antibiotic-related KAB questionnaire was developed by the authors specifically for this study based on expert opinion and relevant literature. An English version of the full questionnaire is provided as Supplementary File 1. Example items include: ‘Antibiotics are effective for viral illnesses such as the common cold or flu.’ (Knowledge); ‘Using antibiotics without a prescription is safe.’ (Attitudes); and 'I have stopped antibiotics before completing the course because I felt better.’ (Behaviors). The content of the questionnaire is designed as follows:

Knowledge: Five items were coded as correct = 1, incorrect or “don’t know” = 0, yielding a total score of 0–5 (higher scores indicate greater knowledge).

- Attitudes: Six statements rated on a 5-point Likert scale (1 = strongly disagree … 5 = strongly agree), with three reverse-coded negative items. Total scores range from 6 to 30; higher scores reflect more appropriate antibiotic-related attitudes.
- Behaviors: Five items were coded dichotomously (0 = inappropriate, 1 = appropriate), producing a total score of 0–5.
- A composite KAB score was calculated by summing the three subscale scores, resulting in a possible range of 6–40.

The five knowledge items were designed to assess distinct, clinically relevant domains, such as the effectiveness of antibiotics against viral infections and the risks associated with non-prescription use. The six attitude items captured different evaluative dimensions, including perceived necessity (e.g. expecting antibiotics for a fever), risk awareness and beliefs about adherence. Finally, the behavior items (five items) assessed concrete practices such as self-medicating with leftover medication and adhering to prescribed durations. These items represent formative components that collectively define the constructs, rather than being redundant indicators of a single trait. This heterogeneous design was intentional, providing a comprehensive overview of public perceptions.

To ensure the scale’s content validity, opinions were sought from three academics specialising in infectious diseases and clinical microbiology. The necessary linguistic and scope adjustments were made to the statements in line with their recommendations.

### Statistical analysis

All analyses were conducted using SPSS version 27.0 and the PROCESS macro version 4.3 (developed by Hayes). Descriptive statistics (means, standard deviations and frequencies) were computed to summarise the characteristics of the sample and the distributions of the scales. The internal consistency of the scales was evaluated using Cronbach’s α. As distributions deviated from normality, group differences were examined using Mann–Whitney U and Kruskal–Wallis tests. Spearman’s rank correlation coefficients were calculated to assess the association between HL and KAB scores.

Multiple linear regression analyses were performed to evaluate predictors of outcomes. All independent variables were entered into the models simultaneously using the ‘enter’ method, adjusting for age group, sex, education, employment and chronic disease status. Mediation analyses were performed using PROCESS Model 4 with 5000 bootstrap samples and 95% bias-corrected confidence intervals to test whether HL mediated the effect of knowledge on attitudes and behaviors. Multicollinearity was checked using tolerance and variance inflation factor (VIF) values. A two-tailed p value of less than 0.05 was considered statistically significant.

### Ethics

The study protocol was approved by the Non-Interventional Clinical Research Ethics Committee of Mardin Artuklu University (Approval No: 2025/9–24, Date: September 9, 2025) and was conducted in accordance with the ethical principles of the Declaration of Helsinki. Informed consent was obtained from all participants in the study.

## Results

### Sample characteristics

A total of 350 participants were analysed, 50% of whom were female, with a mean age of 39.8 ± 12.2 years. The majority had a university education or higher (62%), 19.1% reported having a chronic disease and 64% had used antibiotics at least once in the past year. The results of the HL test showed that 6.9% of people had insufficient HL, 78.6% had problematic HL, and 14.6% had sufficient HL. Sample characteristics are presented in Table 1.

**Table 1:** Socio-demographic and clinical characteristics of the study sample

Characteristic	*n*	%
**Sex**		
Female	175	50.0
Male	175	50.0
**Age group (years)**		
18–34	130	37.1
35–49	120	34.3
50–65	100	28.6
**Education**		
Primary school or less	31	8.9
Secondary school	18	5.1
High school	84	24.0
University or higher	217	62.0
**Employment status**		
Employed	195	55.7
Retired	39	11.1
Unemployed	37	10.6
Housewife	79	22.6
**Chronic disease**		
Yes	67	19.1
No	283	80.9
**Antibiotic use (past year)**		
Never	126	36.0
Once	106	30.3
2–4 times	92	26.3
≥5 times	26	7.4
**Health literacy (HLS-EU-Q6)**		
Inadequate	24	6.9
Problematic	275	78.6
Sufficient	51	14.6
**Total**	350	100

### Sample scale scores and reliability

Mean scores indicated moderate levels of HL, knowledge, attitudes, and behaviors toward antibiotic use. The internal consistency of the HL (α ≈ 0.68) and behavior (α ≈ 0.64) scales was acceptable. In contrast, the knowledge (α ≈ 0.54) and attitude (α ≈ 0.52) subscales exhibited lower Cronbach’s alpha values. While these values are below the conventional threshold of 0.70, they are comparable to those of previous KAB surveys and reflect the diverse nature of the items, which were designed to capture different aspects of antibiotic-related perceptions. For this reason, the knowledge and attitude subscales were retained for subsequent analyses. The descriptive statistics and reliability values of the scales are shown in Table 2.

**Table 2:** Descriptive statistics and internal consistency of health literacy and KAB scales

Scale / Score	*N*	Mean ± SD	Cronbach’s α
Health literacy (0 to 4)	350	2.71 ± 0.44	0.678
Knowledge score (0 to 5)	350	3.13 ± 1.36	0.542
Attitude score (6 to 30)	350	23.9 ± 3.6	0.517
Behavior score (0 to 5)	350	3.05 ± 1.50	0.642
KAB total (6 to 40)	350	30.0 ± 4.9	0.627

*KAB* Knowledge, attitudes, and behaviors

### Group comparisons

In median [IQR] comparisons, the age groups differed in terms of knowledge (*p* = 0.002), behaviors (*p* = 0.028) and total KAB (*p* = 0.040). However, there were no differences in attitudes or HL. By education, participants with university education or higher had higher knowledge, attitudes, and KAB scores (*p* = 0.007, *p* = 0.003, and *p* = 0.005, respectively), while HL and behaviors did not differ. By antibiotic use frequency, increasing use was associated with lower behavior and KAB scores (both *p* < 0.001); attitudes also differed across groups (*p* = 0.007), knowledge was borderline (*p* = 0.052), and HL did not differ. Statistical differences across age and education groups yielded small effect sizes, whereas the frequency of prior antibiotic use showed a medium-to-large impact on behavior scores. Sex-based comparisons showed no significant differences on any scale (all *p* > 0.05). The comparison of HL and KAB scores according to age, gender, education level, and frequency of antibiotic use in the last year is presented in Table 3.

**Table 3:** Group comparisons of health Literacy, knowledge, attitudes, behaviors, and total KAB scores

Variable	HL	Knowledge	Attitudes	Behaviors	Total KAB
Age group 18–34 (*n* = 130)	2.67 [0.50]	3.00 [2.00]	24.00 [5.00]	3.00 [2.00]	30.00 [8.00]
Age group 35–49 (*n* = 120)	2.83 [0.50]	3.00 [1.00]	24.00 [5.00]	3.00 [2.00]	30.00 [6.00]
Age group ≥ 50 (*n* = 100)	2.83 [0.50]	3.00 [2.00]	24.00 [5.00]	4.00 [2.75]	31.00 [8.50]
Age group (KW p value)	*p* = 0.280	*p* = 0.002	*p* = 0.347	*p* = 0.028	*p* = 0.040
Sex Female (*n* = 175)	2.67 [0.50]	3.00 [2.00]	24.00 [5.00]	3.00 [2.00]	30.00 [8.00]
Sex Male (*n* = 175)	2.67 [0.67]	3.00 [2.00]	24.00 [5.00]	3.00 [2.00]	30.00 [7.00]
Sex (MW p value)	*p* = 0.332	*p* = 0.088	*p* = 0.527	*p* = 0.073	*p* = 0.233
Education ≤ High school (*n* = 133)	2.67 [0.67]	3.00 [2.00]	23.00 [6.00]	3.00 [2.00]	29.00 [6.00]
Education ≥ University (*n* = 217)	2.83 [0.50]	3.00 [2.00]	25.00 [5.00]	3.00 [2.00]	31.00 [8.00]
Education (MW p value)	*p* = 0.332	*p* = 0.007	*p* = 0.003	*p* = 0.475	*p* = 0.005
Antibiotic use Never (*n* = 126)	2.67 [0.67]	3.00 [2.00]	24.00 [6.00]	4.00 [2.00]	30.00 [8.00]
Antibiotic use Once (*n* = 106)	2.83 [0.50]	3.00 [2.00]	25.00 [4.25]	3.00 [2.00]	31.00 [6.00]
Antibiotic use 2–4 times (*n* = 92)	2.67 [0.50]	3.00 [2.00]	24.00 [5.00]	3.00 [2.00]	30.00 [6.00]
Antibiotic use ≥ 5 times (*n* = 26)	2.67 [0.66]	2.00 [1.25]	22.00 [5.25]	2.00 [2.00]	25.00 [6.50]
Antibiotic use (KW p value)	*p* = 0.284	*p* = 0.052	*p* = 0.007	*p* < 0.001	*p* < 0.001

Values are presented as median [IQR]. *HL* Health literacy, *KAB* Knowledge, attitudes, and behaviors composite score, *KW* Kruskal–Wallis test, *MW* Mann–Whitney U test

### Correlation analyses

**Fig. 1:**
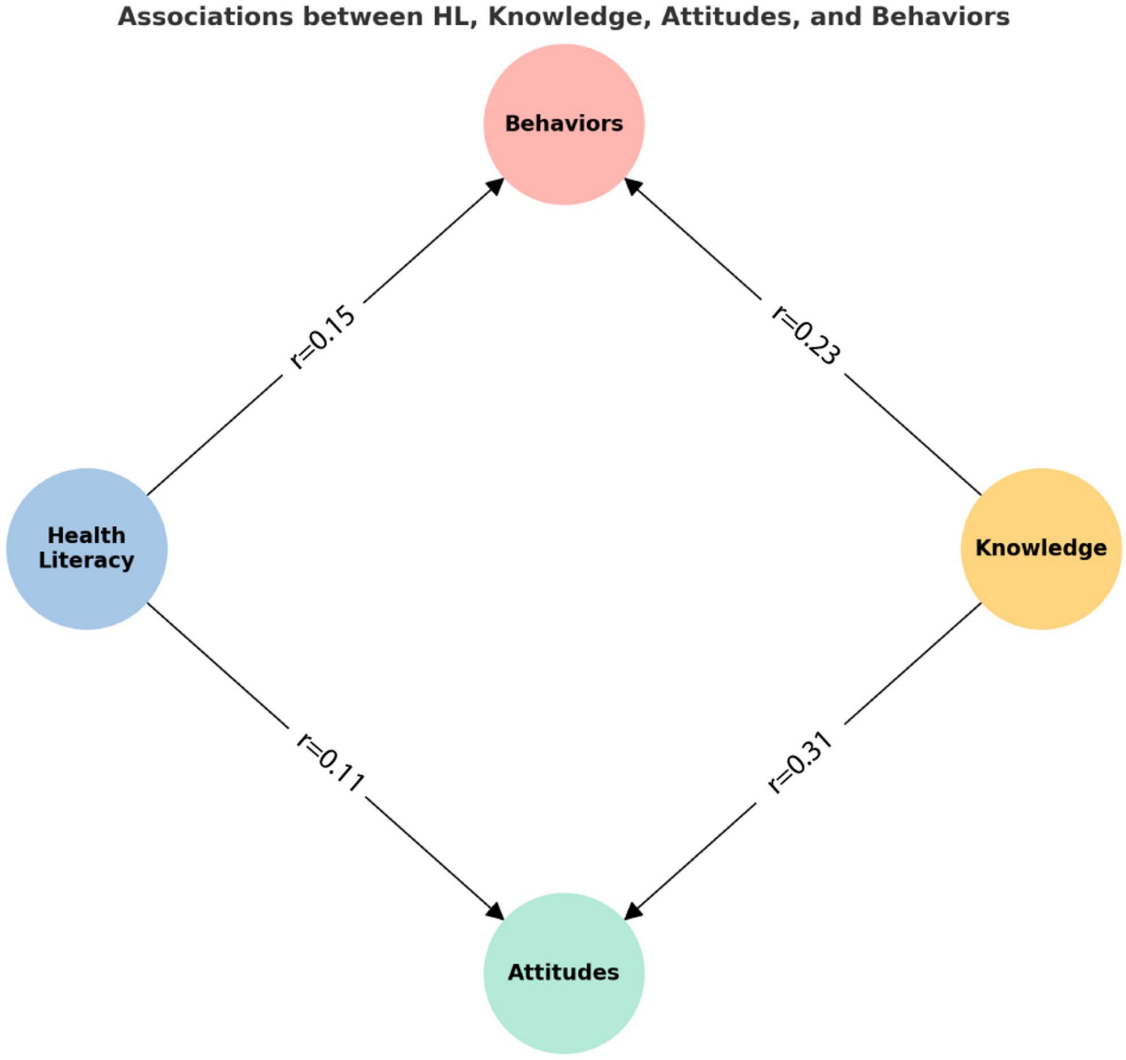
Correlations between HL, knowledge, attitudes, and behaviors. HL was weakly correlated with attitudes (*r* = 0.11) and behaviors (*r* = 0.15), but not with knowledge. Knowledge showed moderate correlation with attitudes (*r* = 0.31) and weak-to-moderate correlation with behaviors (*r* = 0.23)

Spearman’s correlation analyses showed that HL was positively but weakly associated with attitudes (*r* = 0.109, *p* = 0.041), behaviors (*r* = 0.150, *p* = 0.005), and the overall KAB score (*r* = 0.130, *p* = 0.015), with no significant association with knowledge (*p* = 0.348). In contrast, knowledge demonstrated stronger associations: it correlated moderately with attitudes (*r* = 0.307, *p* < 0.001), weak-to-moderately with behaviors (*r* = 0.226, *p* < 0.001), and strongly with the overall KAB score (*r* = 0.553, *p* < 0.001) (Fig. 1).

### Regression analyses

**Fig. 2:**
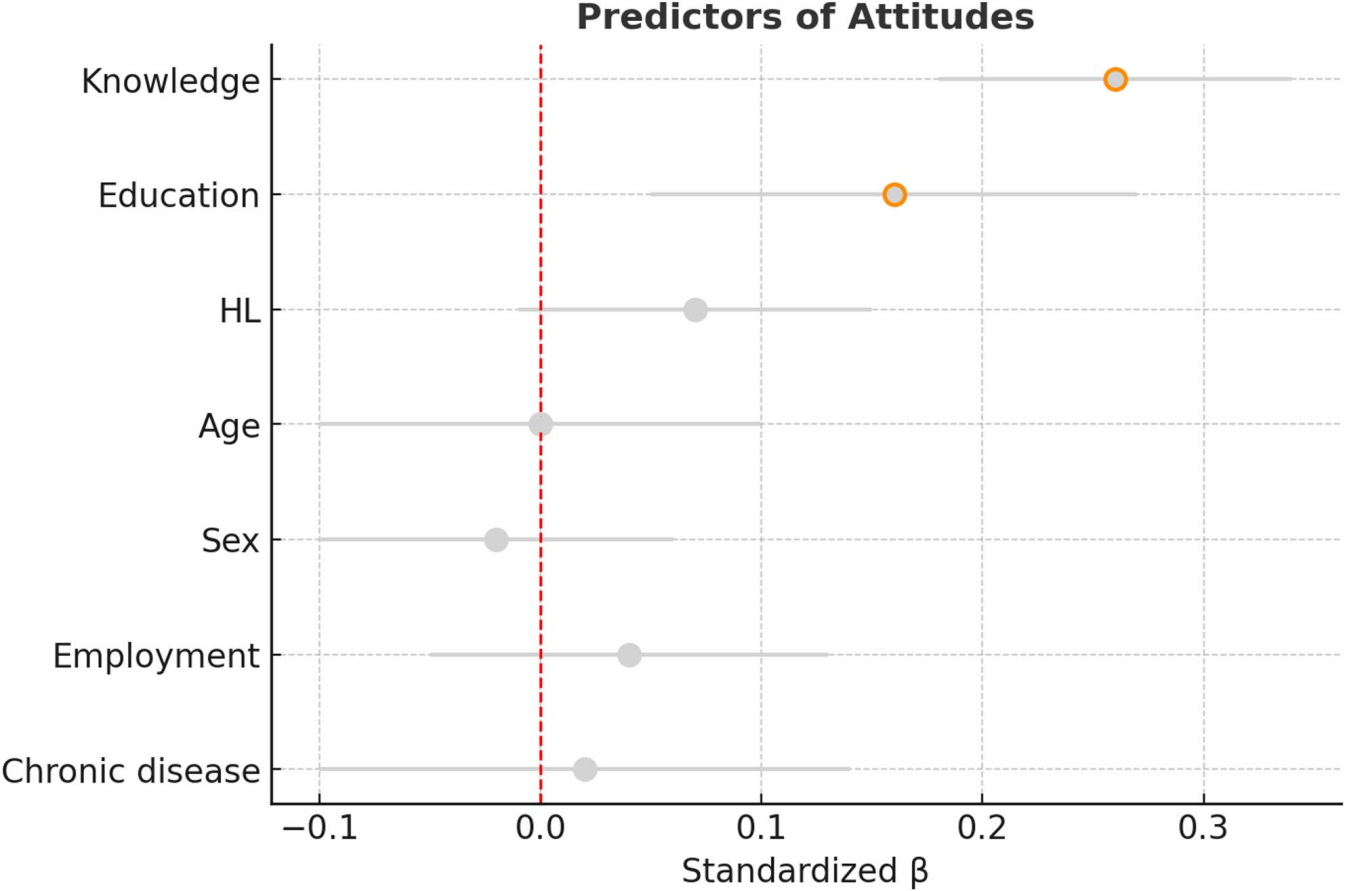
Predictors of attitudes toward antibiotic use. Multiple linear regression model adjusted for sociodemographic factors. Higher knowledge (β = 0.26, *p* < 0.001) and education (β = 0.16, *p* = 0.004) were significant predictors of more appropriate attitudes. HL showed only a marginal effect (*p* = 0.070)

**Fig. 3:**
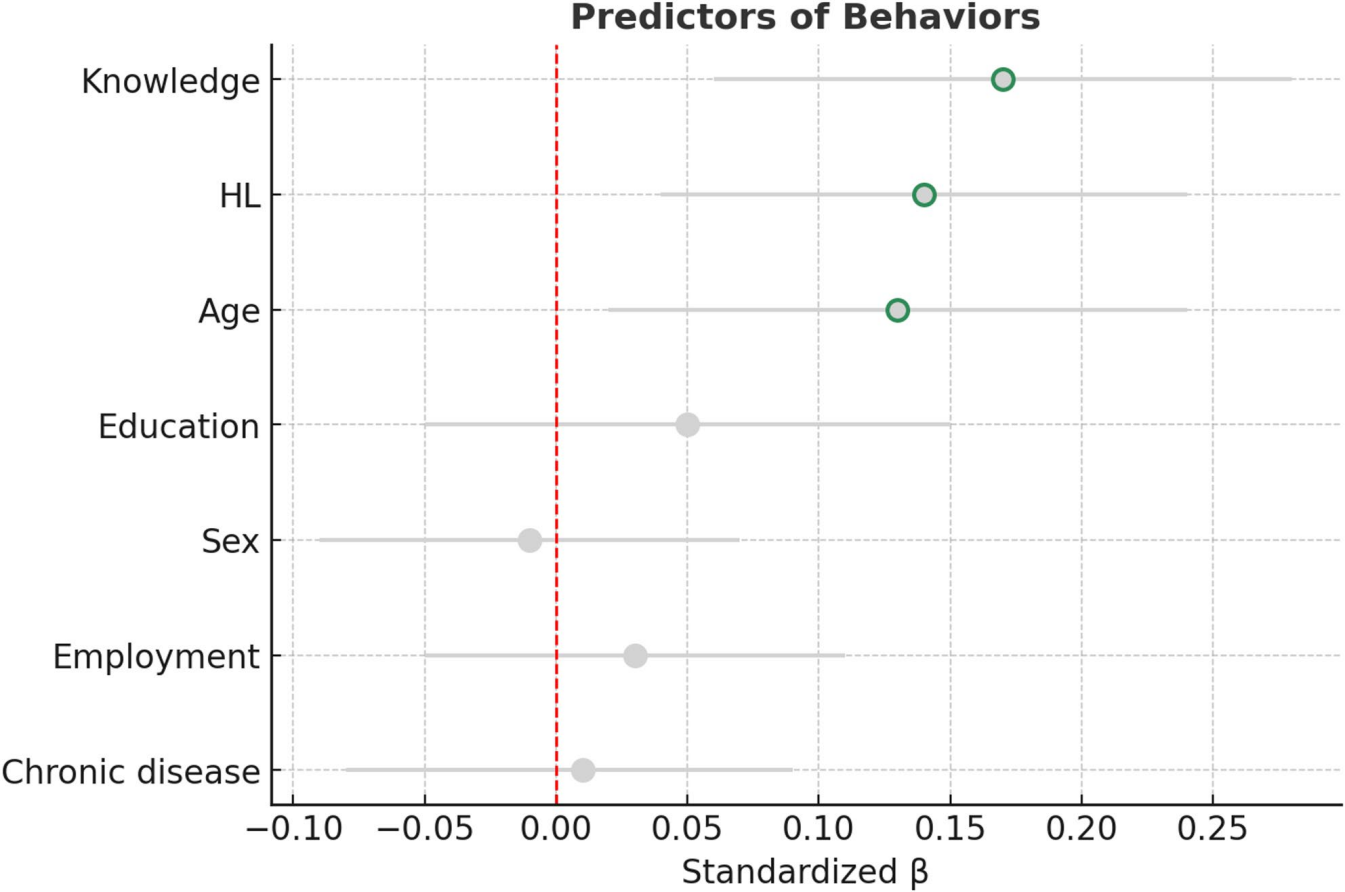
Predictors of behaviors related to antibiotic use. Multiple linear regression model adjusted for sociodemographic factors. Knowledge (β = 0.17, *p* = 0.002), HL (β = 0.14, *p* = 0.008), and age (β = 0.13, *p* = 0.023) were significant predictors of appropriate antibiotic-related behaviors

Multiple linear regression analyses were conducted to identify predictors of attitudes and behaviors toward antibiotic use (Table 4). After adjusting for sociodemographic variables, both knowledge and HL emerged as significant predictors. For attitudes, higher knowledge scores (β = 0.26, *p* < 0.001) and higher education (β = 0.16, *p* = 0.004) were associated with more positive attitudes, while HL showed only a marginal effect (*p* = 0.070) (Fig. 2). For behaviors, both knowledge (β = 0.17, *p* = 0.002) and HL (β = 0.14, *p* = 0.008) were significant predictors, alongside age (β = 0.13, *p* = 0.023) (Fig. 3). Other covariates, including sex and chronic disease status, were not significant predictors in either model. Together, the models explained 12.6% of the variance in attitudes and 8.6% of the variance in behaviors.

**Table 4:** Regression results for attitudes and behaviors

	Predictor	B (SE)	β	95% CI (B)	*p*-value
**Attitudes**	Age (continuous)	0.021 (0.017)	0.071	-0.013, 0.054	0.207
	Male (ref = female)	-0.235 (0.373)	-0.033	-0.969, 0.499	0.529
	Chronic disease (ref = no)	0.147 (0.478)	0.016	-0.794, 1.089	0.758
	Education ≥ University (ref ≤ HS)	1.191 (0.411)	0.161	0.383, 1.999	0.004
	HL	0.769 (0.423)	0.093	-0.063, 1.600	0.070
	Knowledge	0.691 (0.141)	0.260	0.413, 0.969	< 0.001
	**Model R² = 0.126 ** **Adj. R² = 0.111**				
**Behaviors**	Age (continuous)	0.016 (0.007)	0.132	0.002, 0.030	0.023
	Male (ref = female)	-0.213 (0.158)	-0.071	-0.524, 0.098	0.179
	Chronic disease (ref = no)	-0.249 (0.203)	-0.066	-0.648, 0.150	0.221
	Education ≥ University (ref ≤ HS)	-0.101 (0.175)	-0.033	-0.445, 0.243	0.564
	HL	0.477 (0.180)	0.138	0.124, 0.830	0.008
	Knowledge	0.190 (0.060)	0.172	0.072, 0.308	0.002
	**Model R² = 0.086** **Adj. R² = 0.070**				

Multiple linear regression predicting *Attitudes* and *Behaviors* related to antibiotic use. Models adjusted for age (continuous), sex (ref = female), chronic disease, and education (ref ≤ high school). Predictors of interest were health literacy (total score) and knowledge. Values shown as unstandardized coefficients B (SE), standardized β, 95% confidence intervals, and *p*-values

### Mediation and moderation analyses

Mediation (PROCESS Model 4, 5000 bootstraps) was tested to determine whether HL mediated the effects of knowledge on outcomes. The indirect effect was non-significant both for attitudes (indirect effect = 0.036, 95% CI: − 0.18 to 0.26) and behaviors (indirect effect = 0.011, 95% CI: − 0.06 to 0.08). Direct and total effects of knowledge on attitudes (B = 0.73, *p* < 0.001) and behaviors (B = 0.22, *p* = 0.002) remained significant, indicating independent contributions of knowledge and HL.

Moderation analyses (PROCESS Model 1) tested whether HL altered the strength of associations between knowledge and outcomes. No significant interaction effects were found for either attitudes (interaction B = 0.39, *p* = 0.22) or behaviors (interaction B = 0.09, *p* = 0.49), suggesting that the impact of knowledge did not vary across HL levels.

## Discussion

According to our findings, HL level showed a statistically significant, albeit weak, positive correlation with attitudes (*r* = 0.109) and behaviors (*r* = 0.150) related to antibiotics. However, no significant correlation was found between HL and antibiotic knowledge level. On the other hand, the level of antibiotic knowledge was found to be moderately positively correlated with attitudes (*r* = 0.307) and weakly to moderately positively correlated with behaviors (*r* = 0.226). These results suggest that HL has a limited effect on attitudes and behaviors relating to antibiotic use, whereas knowledge of antibiotics is more strongly related to these attitudes and behaviors. Multivariate regression analyses also support these findings. Knowledge level (β = 0.26, *p* < 0.001) was the strongest predictor variable for attitudes towards antibiotics, and education level (β = 0.16, *p* = 0.004) also had a positive effect. HL, on the other hand, showed only a marginally significant effect on attitudes (*p* = 0.070). Regarding behaviors, knowledge (β = 0.17, *p* = 0.002) and HL (β = 0.14, *p* = 0.008) contributed significantly independently, and the age variable (β = 0.13, *p* = 0.023) was also found to be associated with behaviors. These results suggest that knowledge plays a dominant role in shaping attitudes, while HL provides a modest yet independent contribution, particularly in explaining behaviors.

Although HL emerged as a significant predictor of antibiotic-related behaviors, it is important to emphasise that the observed associations were weak (*r* = 0.150) and the regression models explained only a modest proportion of the variance (8.6%). These findings suggest that HL is just one factor influencing how individuals use antibiotics. Other unmeasured variables, such as cultural beliefs, past experiences with healthcare or socioeconomic constraints, are likely to play a substantial role in these outcomes. Nevertheless, HL’s independent significance highlights its potential as a targeted area for public health interventions, even if its individual effect size is limited.

Additionally, the mediation and moderation analyses revealed that HL did not mediate or moderate the relationship between knowledge and attitudes or behaviors. In other words, HL did not act as a mechanism that transforms knowledge into behavior; the contributions of knowledge and HL occurred through independent pathways. The finding that HL contributes to antibiotic-related behaviors independently of knowledge suggests that behavioral decisions may be influenced by factors beyond factual understanding alone. HL involves process-oriented capacities related to interpreting information, engaging with healthcare providers, and evaluating the credibility of health guidance. These capacities may support individuals in navigating treatment recommendations and applying information appropriately, particularly in situations involving uncertainty or competing advice. Thus, HL may facilitate the translation of health information into appropriate actions, even when factual knowledge levels are similar. Therefore, an effective public health strategy must target both knowledge and HL, as neither alone is sufficient.

Consistent with previous studies, our findings suggest that a higher level of knowledge about antibiotic use is associated with more positive attitudes and behaviors [13–15]. Recent work has similarly demonstrated that antibiotic-related attitudes and risk awareness regarding AMR are closely linked to antibiotic use behaviors, sometimes more strongly than factual knowledge alone [16]. The relationship between HL and antibiotic-related KAB is inconsistent in the literature. In Egypt, for example, a strong correlation was found between HL and antibiotic knowledge in the general population (*r* = 0.876; *p* = 0.001) [17]. In a similar study conducted with medical students in Egypt, no difference was found in antibiotic knowledge levels between the HL categories (inadequate, problematic, adequate) [5]. However, some studies have reported that individuals with low HL levels tend to request antibiotics more frequently in viral infection scenarios [18] and that those who used antibiotics in the past year had lower HL scores than those who did not use antibiotics [6].

In the present study, no significant correlation was found between HL and antibiotic knowledge levels. However, a positive correlation was observed between HL and attitudes and behaviors. The lack of a significant association between HL and antibiotic knowledge merits further consideration. From a theoretical perspective, HL may be more closely linked to the evaluation and use of health information than to acquiring specific factual knowledge, particularly in environments where basic antibiotic information is widely available. Contextually, repeated exposure to standardised antibiotic-related messages through healthcare encounters and public health campaigns may reduce variability in knowledge across health literacy levels. Methodologically, the heterogeneous and content-specific nature of the knowledge items used in this study may have limited their sensitivity to variation in general HL. Taken together, these factors suggest that, although conceptually related, antibiotic knowledge and HL may not be strongly coupled in all populations.

Therefore, it can be concluded that the relationship between HL and antibiotic use may vary between populations and sample groups. The inconsistent findings regarding the HL–KAB relationship in different studies may be due to differences in the way the relationship was measured. The KAB questionnaire developed for this study may reveal different results to those obtained using more descriptive awareness scales. These methodological differences, combined with cultural context, may explain the heterogeneity observed in the international literature.

We found that positive attitudes and appropriate behaviors regarding antibiotics increased with age. The literature suggests that age may influence antibiotic use. For instance, a study of the general population in Singapore found that older age groups had higher antibiotic-related KAB scores [14]. Similarly, it has been shown that age in the community sample can modify the relationship between knowledge of antibiotic and AMR and inappropriate use, and that the likelihood of inappropriate use decreases with advancing age [19]. Our findings indicate that older age groups may be more inclined toward prudent patterns of antibiotic use. Nevertheless, the association between age and antibiotic-related behaviors is likely multifactorial, reflecting not only accumulated experience but also differential access to healthcare services, cohort effects, and broader sociocultural determinants.

Non-parametric comparisons revealed significant differences in the age, educational level, and frequency of antibiotic use categories in the past year. The detection of differences favoring knowledge, behavior, and total KAB scores in age groups is consistent with the aforementioned age effect and suggests a tendency towards more cautious use with increasing age. As reported in different populations, our findings show that knowledge, positive attitudes, and appropriate usage behaviors regarding antibiotics increase with level of education [20, 21]. Another noteworthy result indicates that those who used antibiotics more frequently in the past year had lower attitude and behavior scores. This suggests an inverse relationship, indicating that demand-driven use or self-initiated behavior may be more prevalent [19, 22]. Our study found no gender differences in HL or in KAB related to antibiotics. Some studies have found no gender differences in antibiotic KAB scores [23, 24]. However, other studies have found that non-prescription antibiotic self-medication is more common among men [25, 26]. Similar heterogeneous results were found for HL levels according to gender. Data from the Health Literacy Survey in Europe (HLS-EU) study covering many countries show that HL may be slightly higher in women than in men [27], but it has also been reported that results vary across different samples [28].

From a public health perspective, these findings suggest that interventions aimed at improving antibiotic use should go beyond simply increasing factual knowledge and should also incorporate strategies informed by HL. These could include tailored messaging emphasising the risks of AMR in clear, accessible language; simplified prescribing instructions supporting correct antibiotic use; and community-based interventions facilitating informed decision-making in everyday contexts. Therefore, integrating HL principles into antimicrobial stewardship initiatives may enhance their effectiveness by supporting how individuals interpret and apply their knowledge in practice.

### Strengths and limitations

The strengths of our study include the use of pilot testing to ensure measurement quality, a sample size calculation based on power analysis and a stratified quota sampling design to enhance representativeness by reflecting the national distribution of age and gender. Unlike studies based on convenience samples, our design provides stronger external validity.

However, several limitations must also be acknowledged. Firstly, this study was limited by the modest internal consistency observed for the knowledge and attitude subscales (Cronbach’s α < 0.70). However, this was an expected consequence of the scale’s multifaceted design. Unlike scales that measure a single psychological trait, these subscales combine different categories of clinical knowledge and attitudes. Consistent with this content-driven and heterogeneous structure, exploratory or confirmatory factor analysis was not performed, which limits conclusions regarding the underlying factor structure of the instrument. It is important to note that this lower reliability may have led to conservative effect estimates, potentially attenuating the observed correlations between HL and KAB components. Secondly, although the sample was stratified by age and gender to resemble the national distribution, the educational level was higher than in the general population due to the online participation method. Thirdly, the data were self-reported and thus susceptible to recall and social desirability biases. Fourthly, the recruitment source (online vs. in-person) for individual participants was not recorded, preventing a sensitivity analysis that would allow these sub-populations to be compared. Although quota sampling was used to balance demographic characteristics across recruitment modes, the lack of differentiated data limits our ability to assess potential differences in health-seeking behaviors between the two groups. Finally, the cross-sectional design precludes causal inferences. Further longitudinal and interventional studies are required to validate these relationships and investigate causal mechanisms.

## Conclusion

This study has revealed that HL and knowledge of antibiotics influence community attitudes and behaviors in different ways. Knowledge level is the strongest predictor of positive attitudes towards antibiotics, while HL contributes independently to shaping behaviors. In order to promote the rational use of antibiotics in society, antibiotic knowledge should be increased through educational campaigns in schools and among the general public. At the same time, health communication and counselling services should be strengthened to improve HL.

## Supplementary Information

Supplementary Material 1.

## Data Availability

The datasets used and/or analysed during the current study are available from the corresponding author on reasonable request.

## Abbreviations

AMR: Antimicrobial resistance
HL: Health literacy
IQR: Interquartile range
KAB: Knowledge, attitudes, and behaviors
