# Supplementary material for: Association between health literacy and antibiotic-related knowledge, attitudes, and behaviors
Source: BMC Public Health. 2026 Jan 26;26:656. doi: 10.1186/s12889-026-26416-2 (PMC12917959; doi:10.1186/s12889-026-26416-2)
Supplement: Supplementary file 1 — Supplementary Material 1. [file 12889_2026_26416_MOESM1_ESM.docx]

**Antibiotic-Related Knowledge, Attitudes, and Behaviors Questionnaire (English Version)**

1. Antibiotics are effective for viral illnesses such as the common cold or flu.

- True
- False
- I don't know

1. In most febrile illnesses, antibiotics are not necessary.

- True
- False
- I don't know

1. Unnecessary use of antibiotics leads to antimicrobial resistance.

- True
- False
- I don't know

1. Antibiotic resistance means that antibiotics become more effective against microbes.

- True
- False
- I don't know

1. Using antibiotics without a doctor's prescription can cause serious health problems.

- True
- False
- I don't know

1. When I have a fever, I expect the doctor to prescribe antibiotics; if not, I feel disappointed.

- Strongly disagree
- Disagree
- Neutral
- Agree
- Strongly agree

1. Using antibiotics without a prescription is safe.

- Strongly disagree
- Disagree
- Neutral
- Agree
- Strongly agree

1. I think antibiotic resistance will be a serious problem in the future.

- Strongly disagree
- Disagree
- Neutral
- Agree
- Strongly agree

1. I believe that unnecessary use of antibiotics can be harmful.

- Strongly disagree
- Disagree
- Neutral
- Agree
- Strongly agree

1. Even if my symptoms improve, I should complete the antibiotic course as prescribed.

- Strongly disagree
- Disagree
- Neutral
- Agree
- Strongly agree

1. It is appropriate to keep antibiotics at home “just in case”.

- Strongly disagree
- Disagree
- Neutral
- Agree
- Strongly agree

1. I have used leftover antibiotics at home later.

- Yes
- No

1. I have used antibiotics based on someone else’s recommendation (non‑physician).

- Yes
- No

1. I have stopped antibiotics before completing the course because I felt better.

- Yes
- No

1. I have used antibiotics for common cold or flu in the past.

- Yes
- No

1. I have used antibiotics in a dose or duration different from the prescription.

- Yes
- No
